# Outcomes of Lateral Tarsal Strip in Conjunction with a Minimal Skin Muscle Excision Underlying Cauterization in Korean Patients with Involutional Entropion

**DOI:** 10.3389/fsurg.2022.870751

**Published:** 2022-05-12

**Authors:** Hyunkyu Lee, Eunhyang Cha, Sehyun Baek

**Affiliations:** ^1^ Department of Ophthalmology, Korea University College of Medicine, Ansan Hospital, Ansan, South Korea; ^2^ Department of Ophthalmology, Korea University College of Medicine, Guro Hospital, Seoul, South Korea

**Keywords:** involutional entropion, lateral tarsal strip, skin muscle excision, lid laxity, entropion correction

## Abstract

We reviewed the medical records of 53 involutional entropion patients, who underwent lateral tarsal strip (LTS) with a minimal skin muscle excision by cauterization from March 2014 to December 2017, to evaluate the effectiveness and efficiency of LTS in conjunction with a minimal skin muscle excision using cautery in patients with involutional entropion. We evaluated the success rate, complications, recurrence rate, and degree of satisfaction of our technique. Of 53 patients, there were 5 bilateral cases for a total of 58 eyelids. The average of age was 71.2 years old (57–90 years). The average follow-up period was 18 months (12–39 months). The success rate for involutional entropion correction was 98.1% with our technique. There was one mild recurrence case at 7 months. In our study, the average operation time was 20.8 min (15–29 min) for 48 unilateral cases and 27.2 min (20–32 min) for 5 bilateral cases without intraoperative complications. Of 42 responders of patients’ satisfaction questionnaire, 38 patients showed good satisfaction and were willing to recommend the surgery to their acquaintances. The technique of LTS with minimal skin excision with cauterization was effective and provided satisfying outcomes to patients with involutional entropion.

## Introduction

Involutional entropion is the most prevalent form among the various types of entropion. Affected eyelids rotate inward against the cornea and the resultant corneociliary touch causes intolerable ocular irritation and keratopathy (1). Asians may be more predisposed than Caucasians to the development of involutional entropion. This may be due to Asians having a different orbital vector and other orbit biometric parameters (2, 3). Regardless of the ethnic differences about orbital vector or parameters, it is widely believed that horizontal lid laxity, preseptal orbicularis oculi muscle overriding, and inferior retractor dehiscence or weakening have fundamental roles to play in the development of involutional entropion.

The surgical correction of entropion requires an understanding of the basis of the anatomy and pathophysiology for adopting a direct surgical approach in order to correct the anatomic involutional changes (4). Lee et al. emphasized the importance of horizontal laxity in involutional entropion and showed the effectiveness of the lateral tarsal strip procedure in the treatment of involutional entropion (5). Lateral tarsal strip was reported by Tenzel in 1969, and the procedure became prevalent in the late 1970s. As the treatment strategy for involutional entropion developed, researchers started to combine surgical procedures to manage more pathogenic components of involutional entropion. Some case series and comparative studies about combined surgery reported higher success rates and lower recurrence (6–9).

However, there has been no consensus yet on the standard treatment for involutional entropion, and as a result, researchers are introducing various combinational surgical techniques. In this study, we used a lateral tarsal strip procedure in conjunction with a minimal skin muscle excision underlying cauterization in Korean patients with involutional entropion. We evaluated the success rate, intraoperative complication, and recurrence rate. Also, we examined the levels of patients’ satisfaction with the result of our technique through the survey.

## Materials and Methods

### Patients

We reviewed the medical records of 72 involutional entropion patients who visited the ophthalmology department of Korea University Guro Hospital from March 2014 to December 2017. The exclusion criteria included different types of entropion, any kind of previous lower lid surgery, a follow-up period of less than 12 months, and other surgical techniques for involutional entropion treatment rather than lateral tarsal strip surgery in conjunction with a minimal skin muscle excision using cautery. Of 72 patients, 19 were excluded, 11 due to other surgical methods and 8 due to a short follow-up period. After all, a total of 58 lower eyelids of 53 patients met the criteria and were included in the study. The study was approved by the Institutional Review Board of Korea University Medical Center.

We collected data pertaining to demographic information for each subject, the follow-up period, the surgical time, intraoperative and postoperative complications, the surgical success rate, and the recurrence rate. Success was defined as no corneociliary touch in resting through the whole follow-up period. When ciliary contact with cornea was observed, it was defined as undercorrection if the contact was noted within 1 month after the surgery. It was defined as recurrence if the contact was noted after 1 month.

To evaluate the degree of patient satisfaction with the result of our technique, we conducted a survey by questionnaire. The patients were asked, “Are you satisfied with the result of the surgery for involutional entropion?” A 4-score-scale was used to answer: 1 for unsatisfying and want to receive further surgery, 2 for unsatisfying but do not want further treatment, 3 for satisfying but not convinced to recommend to acquaintances, and 4 for very satisfying and willing to recommend to acquaintances.

### Surgical Methods

Surgery was performed under local anesthesia in all patients. Local anesthesia included proparacaine hydrochloride (Alcaine 0.5%, Alcon) and subcutaneous infiltration of the lower eyelid and lateral canthus with half-and-half by volume of lidocaine 2% with 1:100,000 epinephrine and bupivacaine hydrochloride 0.5% solution (Marcaine). In addition, the time it took to complete the procedure was calculated from the moment local anesthetic was administered to the time the incision was closed with tape.

#### Lateral Tarsal Strip (LTS)

First, lateral canthotomy and inferior cantholysis were performed. The lower canthal tendon was made from the lateral lower lid by trimming the mucocutaneous junction, dissecting the anterior lamella, and separating it from conjunctiva. The lower canthal tendon was stretched to the orbital rim so that the surgeon could estimate the amount of shortening required for proper lid tension. The tendon was trimmed and suspended to the periosteum over Whitnall’s tubercle with 4-0 vicryl. After performing minimal skin muscle excision of the lower eyelid with cauterization, the skin of the lateral canthus was then reapproximated, and skin suture was done with 7-0 silk.

#### Minimal Skin Muscle Excision Technique with Cauterization

**Figure 1:**
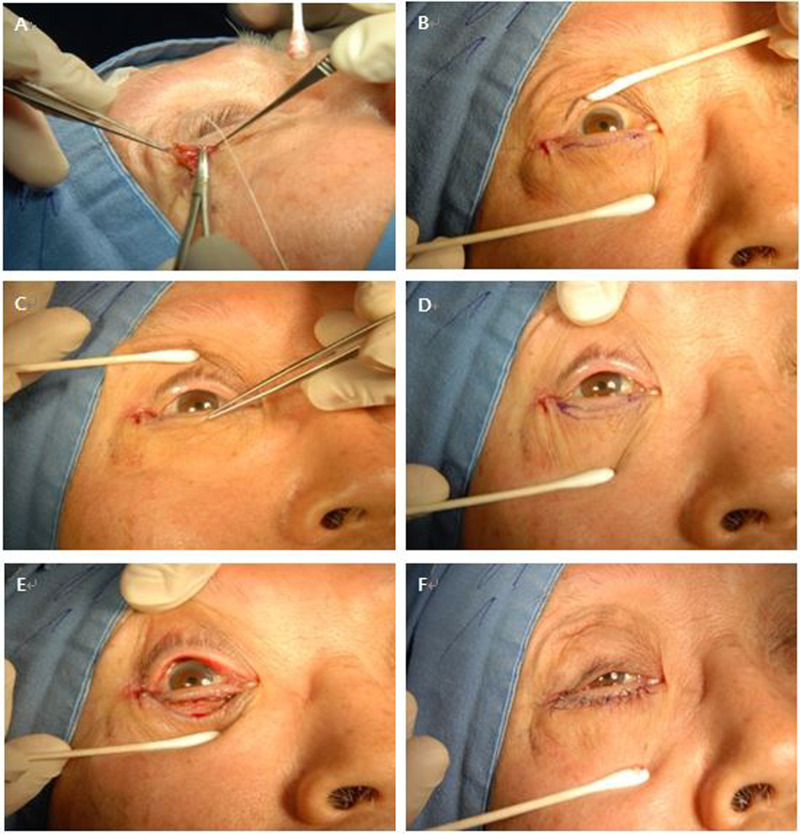
Lateral tarsal strip operation c Skin muscle excision surgery. (**A**) The lower canthal tendon was made from the lateral lower lid by trimming. (**B**) Lateral canthal tendon was fixated to Whitnall’s tubercle, and the subciliary line was drawn 2 mm below the margin of the lower eyelids. (**C**,**D**) The amount of excess skin was measured by pinching with forceps. Then, a crescent-like design was drawn. (**E**) Skin excision was done with westcott scissors, and the overriding orbiculi muscle was removed with cautery. (**F**) Skin was sutured.

Epinephrine: Lidocaine 1:100,000 solution was injected into the lower eyelid. The superior skin incision line was marked with a surgical marking pen 2.0 mm below the lid margin. By pinching the excess skin at the center of the lower lid with a tooth forceps, the skin and muscle removal amounts were measured, and the lower skin incision line was designed in the shape of a crescent. The skin incision was done with an ellman tip along the designed line, and redundant skin was removed with westcott scissors. With bleeding control, the overriding orbicularis oculi muscle was excised with cautery. Finally, skin suture was done with 7-0 black silk (Figure 1).

## Results

**Figure 2:**
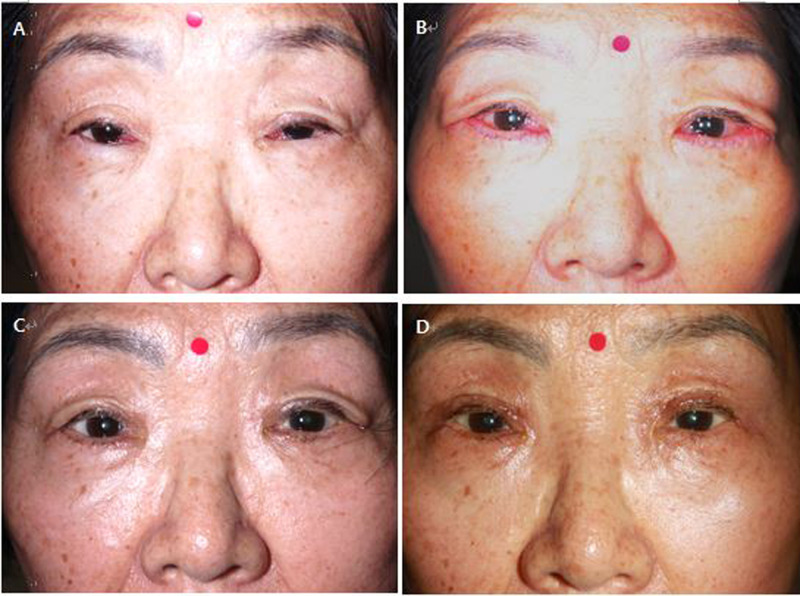
Photographs of a patient who underwent lateral tarsal strip c minimal skin muscle excision with simultaneous cauterization on both lower eyelids. (**A**) Preoperative photograph (**B–D**) Postoperative photographs: 1 week, 1 month, and 6 months after the surgery in each.

Among the 53 patients, 48 (90.6%) were female and the remaining five (9.4%) were male. There were 5 bilateral cases for a total of 58 eyelids. The average age was 71.2 years (range, 57–90 years). All patients received LTS with minimal skin muscle excision using cautery, and the patients, except only one (98.1%), were noted to have successful correction of entropion. The average follow-up period was 18 months (range, 12–39 months) (Table 1). Figure 2 presents preoperative and postoperative (1 week, 1 month, and 6 months) photographs of a patient who underwent lateral tarsal strip c minimal skin muscle excision with simultaneous cauterization on both lower eyelids, and it shows a good result.

**Table 1:** Patients’ characteristics.

	Overall
Patients (*n*/eyelids)	53/58
Age (years, ±SD^a^) (range)(SD)	71.2 ± 9.99 (57–90)
Sex (Female/Male)	48 (90.6)/5 (9.4)
Laterality (Right/Left)	30/28
Follow-up (months, ±SD^a^) (range)	18 ± 6.19 (12–39)
Success rate	98.1%
Surgical time (min, ±SD^a^) (range)	
For unilateral cases (*n *= 48)	20.8 ± 3.46 (15–29)
For bilateral cases (*n* = 5)	27.2 ± 4.42 (20–32)

*^a^* *SD, Standard deviation.*

One mild recurrent entropion case was noted postoperatively at 7 months. A couple of cilia touched the cornea in slit examination. However, the patient did not suffer from ocular discomfort, so further corrective surgery was not taken. In all cases, the surgery was performed without intraoperative complications. Postoperative complications were minimal. Two patients complained of bruises and lid swelling that lasted more than a week following surgery, and one patient had mild ectropion. However, with conservative management, such as massage, all three patients improved within a month following surgery. In the majority of cases, the patients complained of periorbital bruising and eyelid swelling that resolved spontaneously over a few weeks.

A questionnaire on patient satisfaction levels was prepared when the study was planned, and the response was collected in various ways such as e-mail, telephone, and face-to face in clinic. In the questionnaire, 42/53 (79.2%) patients responded. Of the non-responders, one patient died and three refused, and the remaining 7 could not be reached. Of 42 responders, 38 reported a score of 4, and 4 reported a score of 3 (Table 2).

**Table 2:** Questionnaire response of satisfaction with LTS c skin muscle excision.

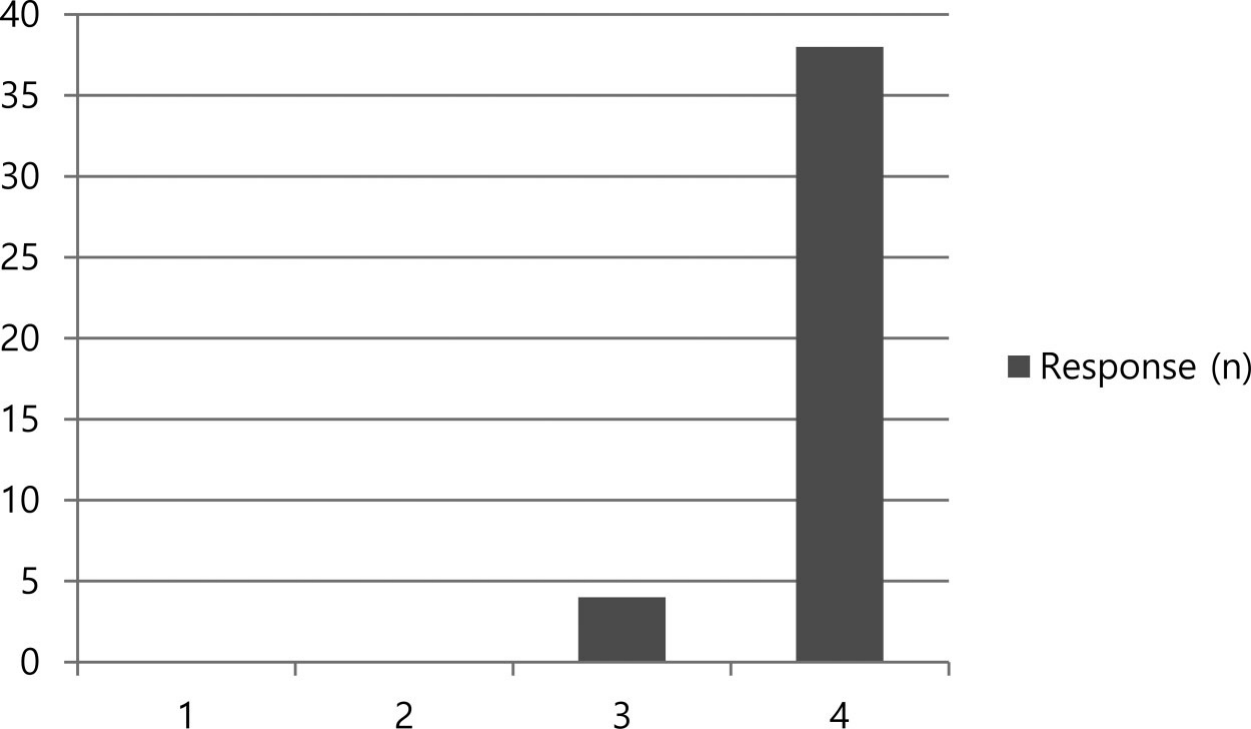

## Discussion

The lateral tarsal strip procedure was introduced by Tenzel in 1969, and it was popularized for more common use by Anderson and Gordy in 1979 (10). Rougraff et al. (6) reported that the lateral tarsal strip procedure for involutional entropion demonstrated a 78% success rate. With diverse combinational techniques, the researchers reported that they could treat involutional entropion with higher success rates ranging from 83 to 100%, although the follow-up period varied from 6 to 45 months (11–15). Various strategies for correcting involutional entropion have also been presented. The result of correcting entropion with a success rate of 97.3% was reported with three small incision techniques (16), and a success rate of 94.1% was reported using two-paired mini-incisional entropion surgery (17). Reinforcement of the lower eyelid retractors with transconjunctival buried sutures was performed through a mini-incision in 46 patients, and it reported a 93.5 percent success rate (18). Furthermore, a 93.1% success rate was reported in a study of lateral tarsal strip with everting sutures (19), S.C. Lee et al. (20) reported good outcomes in Asian involutional entropion using a new procedure called lateral tarsoligamentous sling with a modified lateral tarsal strip. Only 0.59% recurrence was reported after Pretarsal orbicularis oculi muscle tightening with skin flap excision (21). In comparison with the procedures that have lately been reported, with the technique of LTS with minimal skin muscle excision with cauterization, success was achieved in 62 (98.2%) eyelids, while recurrence occurred in 1 (1.8%) eyelid. The surgical outcome of our method was comparable to those of other promising surgical techniques for involutional entropion.

In this study, we tried to reduce recurrence by combining the LTS procedure with the skin muscle excision technique, capable of addressing the overriding orbicularis oculi muscle and vertical laxity of lower eyelids. Minimal excision of the orbicularis oculi muscle “with cauterization” is handy and useful, having dual effects of overriding orbicularis oculi muscle removal and of fibrotic scar formation, which serves as a barrier against the relapse of muscle overriding. We excised the “minimal” amount of the orbicularis oculi muscle to avoid overcorrection and ectropion occurrence. There was no postoperative complication of ectropion in our study.

Theoretically, it would be ideal to settle all major components of involutional entropion, which have known to show horizontal lid laxity, preseptal orbicularis oculi muscle overriding, and inferior retractor dehiscence. Although our procedure deals only with the former two of the three causative components, it still shows an effective outcome. If we had added another procedure to repair inferior retractor dehiscence, it would have delayed operation time significantly, while preventing only one recurrence case out of 53 cases. Practically, combining procedures addressing just two components of involutional entropion might be the most efficient treatment strategy in the consideration of effectiveness over other efforts.

The advantages of our combination surgery are its simplicity and short operation time. Nakauchi and Mimura (13) reported that their Jones procedure with a modified Hotz operation lasted 33.4 min. Hoda et al. (22) found that the operation time ranged from 20 to 40 min in the Wies procedure group and from 20 to 45 min in the Jones procedure group. In another study, the authors reported that 22.4 ± 5 min was required to perform their surgical technique, which consisted of posterior layer advancement of the lower eyelid retractor and transcanthal canthopexy procedures (23). For the orbicularis oculi muscle procedure, the mean operation time was 37.6 min (24). In our study, the average operation time was 20.8 min (range, 15–29) for 53 unilateral cases and 27.2 (range, 20–32) minutes for 5 bilateral cases without intraoperative complications. Our method is quick. This may be due to the fact that there is no need to dissect the skin or muscle. A simple operation guarantees less intraoperative complication and faster recovery.

Our study provides the surgical outcome of LTS with skin muscle excision performed by a single surgeon (S.B.). All patients received a standardized procedure, and patients treated with different surgical methods were excluded. We did not classify involution entropion patients on the basis of preoperative evaluation, and some authors might dispute the fact that our surgical outcome may vary according to patient severity or major problematic components of entropion. However, we believe that our procedure has an advantage in terms of a wide range of applicability. In both LTS and skin muscle excision techniques, the amount of correction for involutional entropion can be tailored according to patient status. For LTS, the surgeon can estimate the amount of tendon shortening required for proper lid tension. For skin muscle excision, the operator can measure the eyelid excess to determine the extent of the excision while observing the everting effect on the eyelash.

In the questionnaire, patients who underwent LTS with minimal skin muscle excision with cauterization were generally satisfied. The overall satisfaction levels of the patients reflect that our procedure is effective for involutional entropion and can free patients from ocular irritation.

The limitations of this study are the small number of patients enrolled and the lack of a long-term outcome of our procedure. Some techniques for involutional entropion such as Quickert suture seemed to be excellent for the immediate repair of entropion, but the effect was temporary and the condition recurred in a large percentage of the patients (25). The average follow-up period of this study was 18 months, which was not enough to evaluate the long-term result of the surgery. However, we have practised our technique for years, and we have encountered few patients with recurrence. In the future, we need to verify the prolonged effect of the LTS procedure with skin muscle excision for involutional entropion in a larger group.

We suggest that our procedure is quick and provides a satisfying outcome to patients with involutional entropion. In addition, the technique seems to provide a long-lasting effect for involution entropion correction.

## Data Availability

The original contributions presented in the study are included in the article/supplementary material, and further inquiries can be directed to the corresponding author/s.
